# A systematic review verified by bioinformatic analysis based on TCGA reveals week prognosis power of CAIX in renal cancer

**DOI:** 10.1371/journal.pone.0278556

**Published:** 2022-12-21

**Authors:** Zikuan Zhang, Bo Wu, Yuan Shao, Yongquan Chen, Dongwen Wang

**Affiliations:** Basic Medicine of Shanxi Medical University, Taiyuan, China; University of Florence, ITALY

## Abstract

**Background:**

Carbonic anhydrase IX (CAIX) protein has been correlated with progression and survival in patients with some tumors such as head and neck carcinoma. But renal cell carcinoma is an exception. The prognostic value of CAIX in RCC used to be associated with patients’ survival according to published works. This study aimed to rectify the former conclusion.

**Methods:**

This study was registered in PROSPERO (CRD42020160181). A literature search of the PubMed, Embase, Cochrane library and Web of Science databases was performed to retrieve original studies until April of 2022. Twenty-seven studies, including a total of 5462 patients with renal cell carcinoma, were reviewed. Standard meta-analysis methods were used to evaluate the prognostic impact of CAIX expression on patient prognosis. The hazard ratio and its 95% confidence interval were recorded for the relationship between CAIX expression and survival, and the data were analyzed using Stata 11.0. Then we verify the meta-analysis resort to bioinformatics (TCGA).

**Results:**

Our initial search resulted in 908 articles in total. From PubMed, Embase, Web of Science electronic and Cochrane library databases, 493, 318 and 97 potentially relevant articles were discovered, respectively. We took the analysis between CA9 and disease-specific survival (*HR = 1*.*18*, *95% CI*: *0*.*82–1*.*70*, *I*^*2*^ *= 79*.*3%*, *P*<*0*.*05*), a subgroup then was performed to enhance the result (*HR = 1*.*63*, *95%CI*: *1*.*30–2*.*03*, *I*^*2*^ *= 26*.*3%*, *P = 0*.*228*); overall survival was also parallel with the former (*HR = 1*.*13*, *95%CI*: *0*.*82–1*.*56*, *I*^*2*^ *= 79*.*8%*, *P*<*0*.*05*), then a subgroup also be performed (*HR = 0*.*90*, *95%CI*:*0*.*75–1*.*07*, *I*^*2*^ *= 23*.*1%*, *P = 0*.*246*) to verify the result; the analysis between CAIX and progression-free survival got the similar result (*HR = 1*.*73*, *95%CI*:*0*.*97–3*.*09*, *I*^*2*^ *= 82*.*4%*, *P*<*0*.*05*), we also verify the result by subgroup analysis (*HR = 1*.*04*, *95%CI*:*0*.*79–1*.*36*, *I*^*2*^ *= 0*.*0%*, *P = 0*.*465*); at last the relationship between CAIX and recurrence-free survival got the same result, too (*HR = 0*.*99*, *95%CI*: *0*.*95–1*.*02*, *I*^*2*^ *= 57*.*8%*, *P = 0*.*050*), the subgroup’s result was also parallel with the former (*HR = 1*.*01*, *95%CI*: *0*.*91–1*.*03*, *I*^*2*^ *= 0*.*00%*, *P = 0*.*704*). To validate our meta-analysis, we took a bioinformatic analysis based on TCGA database, survival curve between low and high CAIX expression in four endpoints (DSS, OS, PFI, DFI) have corresponding P value (DSS:*P = 0*.*23*, OS:*P = 0*.*77*, PFI:*P = 0*.*25*, DFI:*P = 0*.*78*).

**Conclusions:**

CAIX expression in patients with RCC is an exception to predict tumor survival. Both low CAIX expression and high expression are not associated with survivals in RCC patients.

## Introduction

Renal neoplasms are one of the most common solid cancers with fast increasing incidence [1]. In 2020, there were 73,750 new renal tumor cases and 14,830 deaths as a result of renal tumors in the United States [2]. In renal tumor, hypoxic state and necrosis often occur, hypoxia is an independent prognostic factor of poor outcome in patients [3] and weakens the efficacy of standard treatment modalities, such as surgery, chemotherapy, and radiotherapy [4–6]. Therefore, many strategies were investigated to measure or quantize tumor hypoxia to predict treatment outcome and to overcome or target tumor hypoxia with newly designed treatments [7–10].

The identification of tissue-based renal cell carcinoma (RCC) biomarkers may assist in predicting post-operative disease progression and response to adjuvant therapy [11]. Carbonic anhydrase Ⅸ (CAIX or CA9), also known as MN protein, has been investigate as a prognostic biomarker in pre and post-operative RCCs [12]. CAIX is expressed at high level in RCC compared to normal kidney tissues [13], and is thought to be a good candidate tissue-based biomarker. Its main function is to maintain the balance between intracellular and extracellular pH by the reaction: CO_2_+H_2_O = HCO_3_^-^+H^+^, thereby generating an acidic extracellular microenvironment [14, 15]. As a hypoxia-related protein, high CAIX expression relates to corresponding hypoxic status in other tumors such as head and neck carcinoma [16]. Hypoxic areas have stronger CAIX expression because of hypoxia inducible factor (HIF) stabilization [14]. Numerous studies have evaluated CAIX as a biomker of prognosis in RCC with mixed conclusions. High CAIX expression has been reported to be associated with good prognosis [17–26], and higher objective response rates in IL-2-treated patients [27]. Other studies reported no correlation between CAIX expression and RCC prognosis [28, 29]. In several studies of ovarian cancer [30–33], treatment-refractory tumors display expression of the hypoxia-induced CAIX and are associated with cancer progression and poor clinical outcome.

Due to these conflicting results, we performed the meta-analysis to determine whether CAIX can be used as a prognostic marker in RCC.

## Materials and methods

### Literature search, eligibility criteria and data extraction

A literature search of original articles on the prognostic role of CAIX in RCC was conducted on the PubMed, Embase, Cochrane library and Web of Science databases using keywords: renal or kidney, cancer or carcinoma or tumor or neoplasm, “Carbonic anhydrase Ⅸ” or “CAIX” or “CA9”, and prognosis or survival. Articles published until April 2022 were included in the search. Inclusion criteria were used for the analysis: (1) diagnosis of RCC confirmed by histopathology and (2) CAIX level detected by immunohistochemistry (IHC) on primary RCC tissue. The number of patients in a given study was not an exclusion criterion. The report with the largest number of patients was included in this study when the same group based on a similar patient cohort were reported by multiple papers.

The data, extracted by SY and WB, included the basic information of the study (first author, publication year, country and case number), tumor characteristics (tumor stage and grade), CAIX cut-off value (CAIX was divided into high expression group and low expression group according to a certain value), and survival outcome (CAIX expression-related survival).

### Quality assessment

Two independent reviewers assessed the quality of all included studies using the Newcastle-Ottawa Quality Assessment Scale for cohort studies (available at: http://www.ohri.ca/programs/clinical_epidemiology/oxford.asp) (S1 Table). The quality assessment score is based on two independent reviewers’ careful reading, with a third reviewer introduced if the assessment score is different. The study quality evaluation was divided into three categories: (1) cohort selection, (2) cohort comparability, and (3) outcome determination. A total of eight components included. The study received one point if the selection and outcome categories had high quality characteristics. The entire points were then totaled together, with higher scores indicating higher methodological quality.

### Publication bias

For analyzing and measuring publication bias, funnel plots, Egger’s test, and Begg’s test were in use respectively.

### Statistical analysis

Hazard ratios (HRs) with 95% confidence intervals (CIs) were employed to depict the impact of CAIX expression on kinds of survivals. Due to the fact that most of the data in the included studies were in the form of hazard ratio, we did the same for our study as well. Compared to odds ratios, hazard ratios are more accurate because they account for time. It is also simple to interpret HR value: When HR > 1, factors with high expression pose a risk, otherwise it acts as a protective factor (HR<1). As long as HRs and 95% CIs appeared in the studies, this data were extracted directly for analysis. If the data was absent, the HRs and 95% CIs from the Kaplan-Meier survival curves could be calculated through the Engauge Digitizer program (version 4.1), then the survival rate was extracted from the curves to get the HR (SE) [34]. The data was imported into STATA 11.0 software for analysis. In addition, adopted Q tests and I^2^ metrics to appraise the heterogeneity of the studies. The significant heterogeneity of the analyzed data necessitated the use of a random-effects model [35]. A fixed-effects model was performed when no heterogeneity was shown among the studies. The fixed effect model should be exercised on the premise of small heterogeneity between studies. Statistical tests (all bilateral identified) considered P<0.05 as significant difference.

### TCGA dataset

Clinical information for RCC was sourced from The Cancer Genome Atlas (TCGA) data portal. 588 cases with the available clinical information were included.

## Results

### Study selection and characteristics

**Fig 1:**
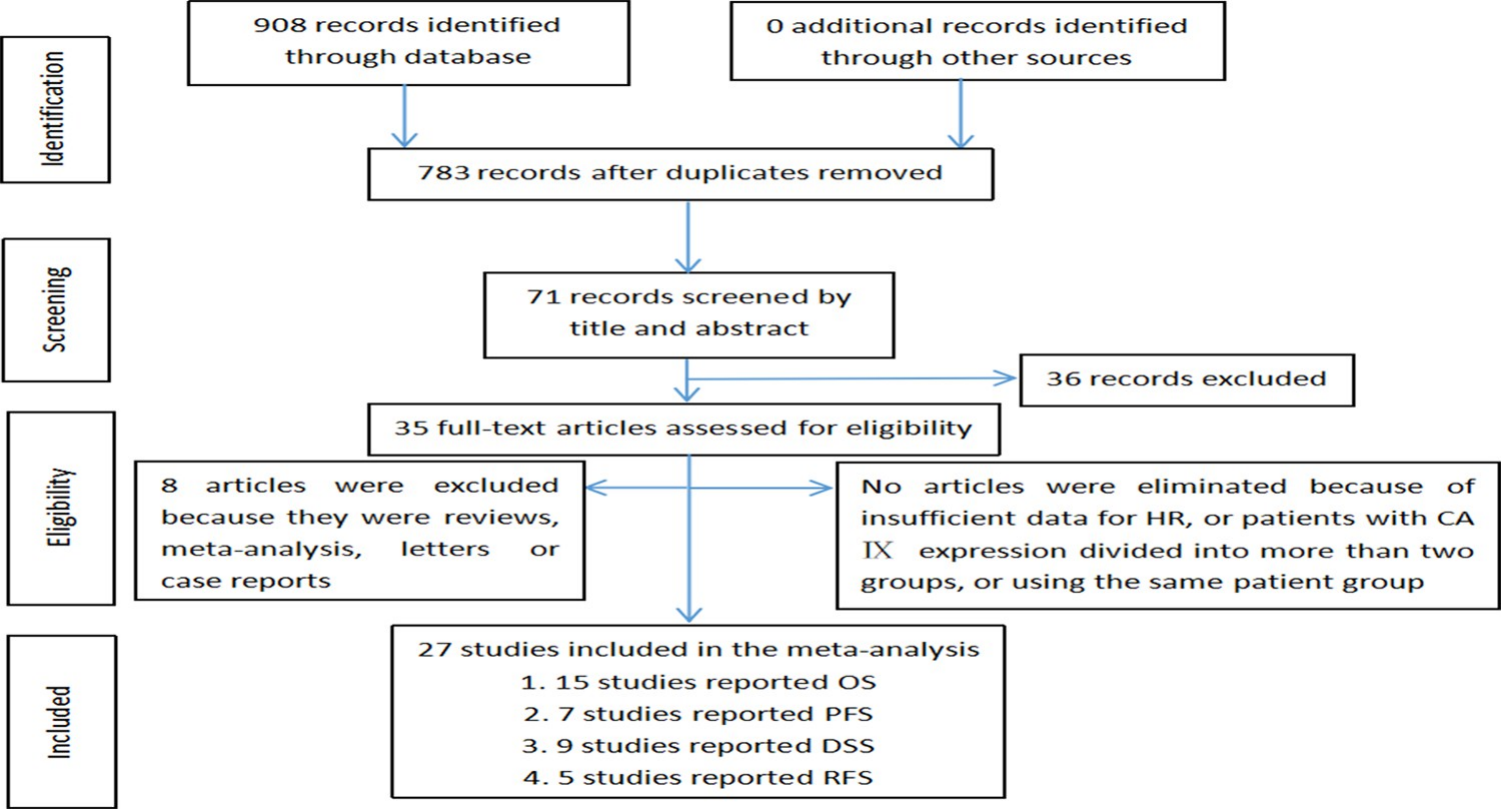
Flow chart of study selection.

Our initial search resulted in 908 articles in total. From PubMed, Embase, Web of Science electronic and Cochrane databases, 493, 318 and 97 potentially relevant articles were discovered, respectively. Clicked the "Remove duplication" option in Endnote software to exclude 125 duplicate papers. Based on the titles and abstracts identified, 71 papers considered eligible for the assessment of the prognostic value of CAIX status in patients with RCC. Our study examined 27 research [17–26, 36–52], 12 of which [37–47, 52] were new articles that published in the previous eight years (after 2014) to assess the connection between CAIX expression and prognosis in RCC patients. Fig 1. depicted the selecting procedure.

Our meta-analysis involved 5,462 patients. Of these studies, 9 were evaluated through multivariate analysis and the other assessed with univariate analysis. Disease-specific survival (DSS) was reported in 9 studies, while in 15, 7, and 6 investigations, overall survival (OS), progression-free survival (PFS), and recurrence-free survival (RFS) were mentioned, respectively. Table 1 illustrated the assessed quality and clinical characteristics of each of those studies, and S2 Table revealed the quality assessment results of each included study in detail.

**Table 1:** Basic characteristics of included studies and quality assessment.

First author	Year	Country	Case	Cut-off value	Survival outcome	Survival analysis	Quality assessment
Grant D. Stewart	2014	UK	45	N/A	OS	Multivariate	7
Inkeun Park	2015	Korea	120	0.475	OS、PFS	Multivariate	6
N.A. Gorban	2016	Russia	60	0.85	DSS	Univariate	7
E.Jason Abel	2014	USA	216	0.45	RFS	Univariate	8
Sung Han Kim	2017	Korea	350	H score	RFS、OS、DSS	Univariate	8
E. Lastraioli	2019	Italy	30	N/A	CSS、RFS	Univariate	8
Bulent Cetin	2015	Turkey	45	N/A	OS、PFS	Univariate	7
A.Ingels	2016	Netherlands	143	N/A	RFS	Univariate	7
Franziska	2018	German	890	N/A	OS、RFS	Univariate	7
S.Chow	2016	UK	100	N/A	OS	Univariate	6
Wenjuan Yu	2017	China	42	N/A	OS	Univariate	7
Karim Chamie	2015	USA	813	0.85	OS	Multivariate	8
Atkins M	2005	USA	66	0.85	OS	Univariate	6
Biswas S	2012	UK	112	N/A	OS	Multivariate	7
Bui MH	2003	USA	321	0.85	DSS	Univariate	6
Choueiri TK	2012	USA	133	0.85	PFS	Univariate	6
Dornbusch J	2013	Germany	42	N/A	OS, PFS	Multivariate	6
Dudek AZ	2010	USA	47	0.85	OS,PFS	Univariate	7
Kim HS	2011	Korea	56	0.85	PFS	Univariate	8
Klatte T	2007	USA	357	N/A	DSS	Multivariate	7
Muriel LC	2012	Spain	135	0.85	PFS,OS	Univariate	6
Patard JJ	2005	France, USA	100	0.85	DSS	Multivariate	7
Phuoc NB	2008	Japan	122	Score 4	DSS	Multivariate	6
Sandlund J	2007	Sweden	228	0.1	DSS	Multivariate	7
Soyupak B	2005	Turkey	67	0.5	OS	Univariate	6
Zerati M	2013	Brazil	95	N/A	OS	Univariate	7
Zhang BY	2013	USA	730	0.85	DSS	Univariate	6

### CAIX expression level was not related to DSS in RCC

**Fig 2:**
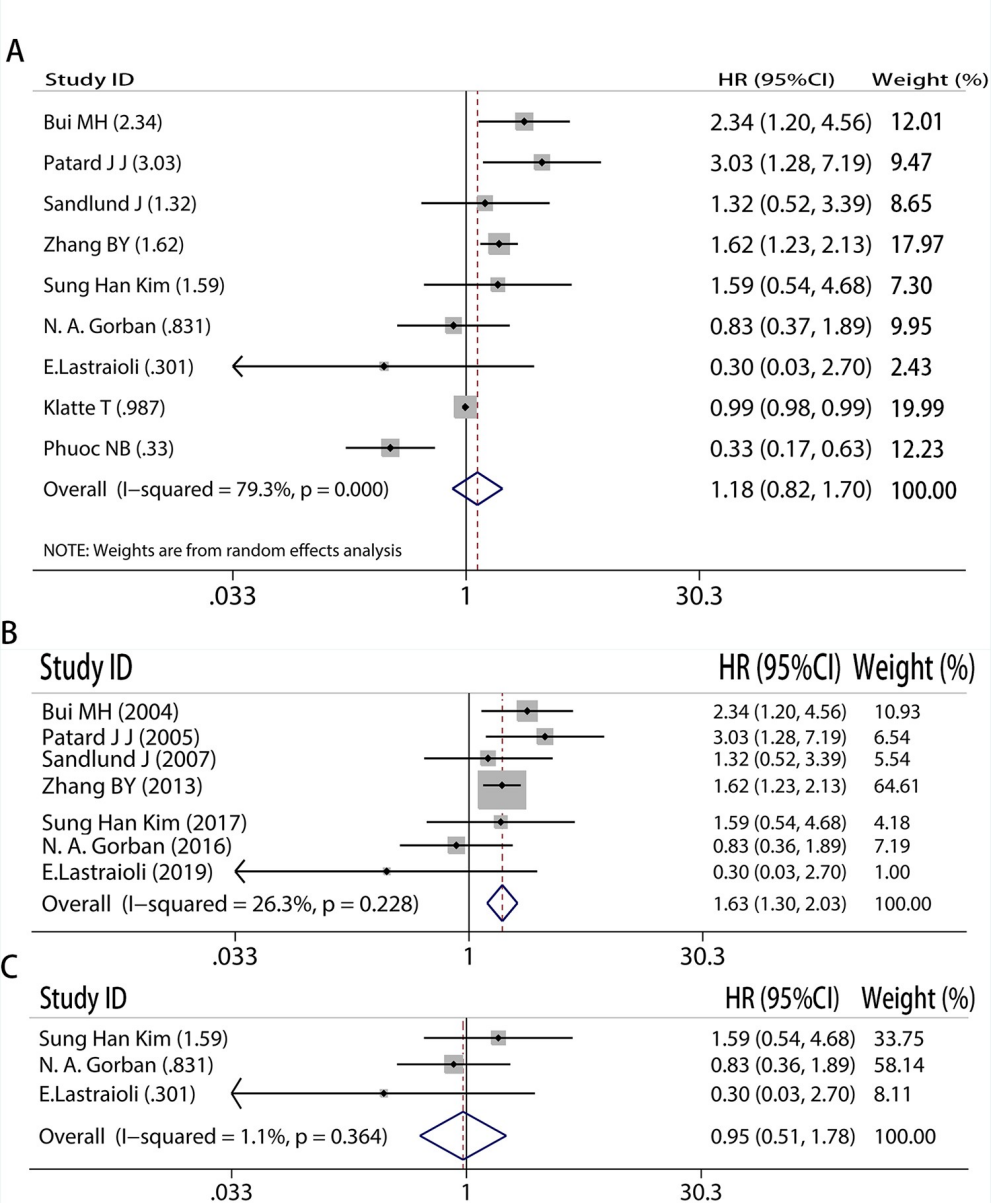
Meta-analysis of CAIX expression and disease-specific survival on A, all inclusion studies; B, by excluding the low quality score studies (quality score≤6); C, only including new studies.

First, we conducted an analysis that included all trials and discovered that CAIX expression was not linked to DSS (HR = 1.18, 95% CI: 0.82–1.70, I^2^ = 79.3%, P<0.05, Fig 2A). However, enormous heterogeneity prompted us to take a subgroup to re-verify the conclusion. Subgroup analysis of high-quality literature showed that high expression of CAIX was associated with longer survival. (HR = 1.63, 95%CI: 1.30–2.03, I^2^ = 26.3%, P = 0.228, Fig 2B). At last we had subgroup which only including new studies (HR = 0.95, 95%CI: 0.51–1.78, I^2^ = 1.1%, P = 0.364, Fig 2C), the result did not associate to DSS as before.

### CAIX expression level can not predict OS in RCC

**Fig 3:**
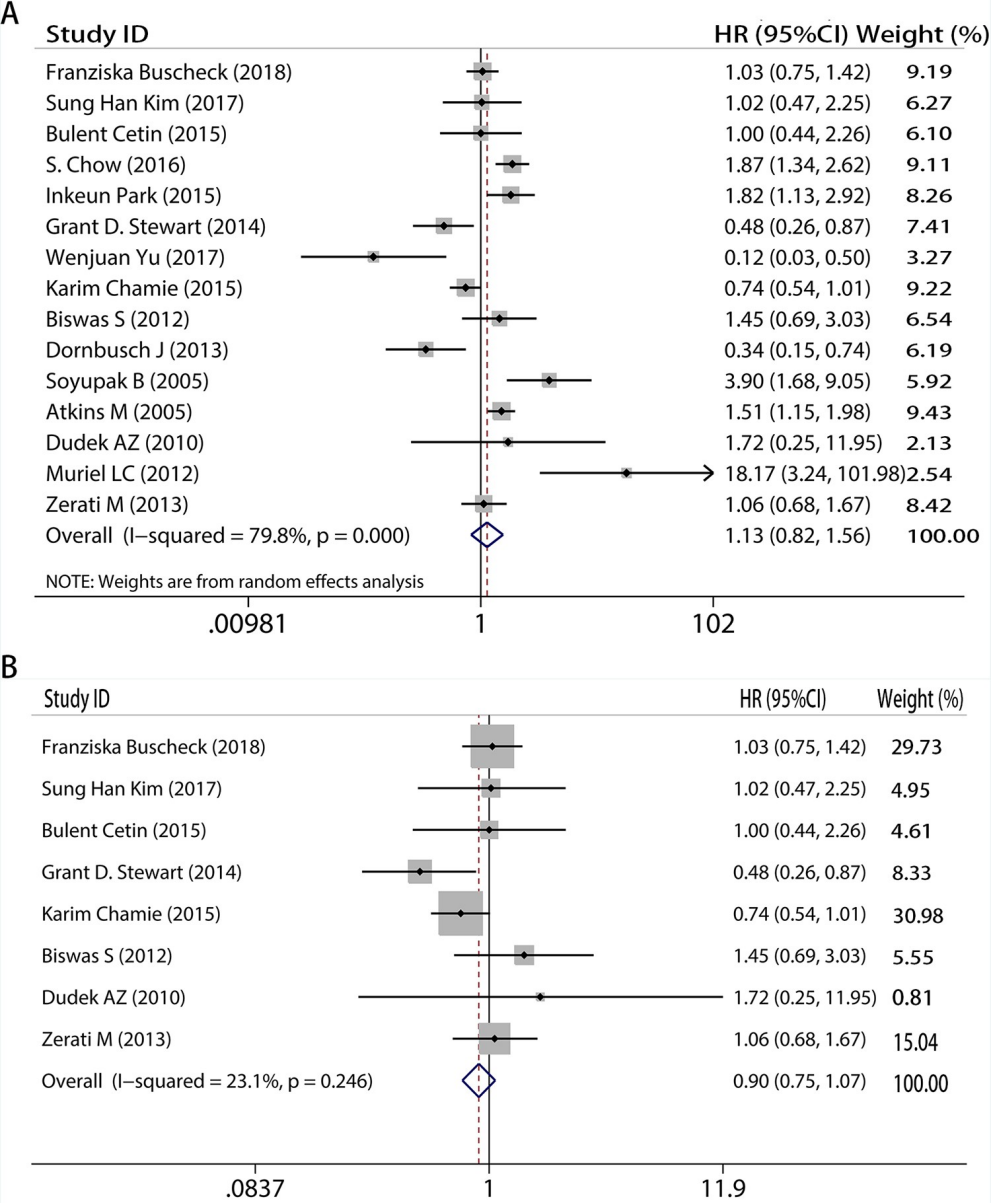
Meta-analysis of CAIX expression and overall survival on A, all inclusion studies; B, by excluding the low quality score studies (quality score≤6).

**Fig 4:**
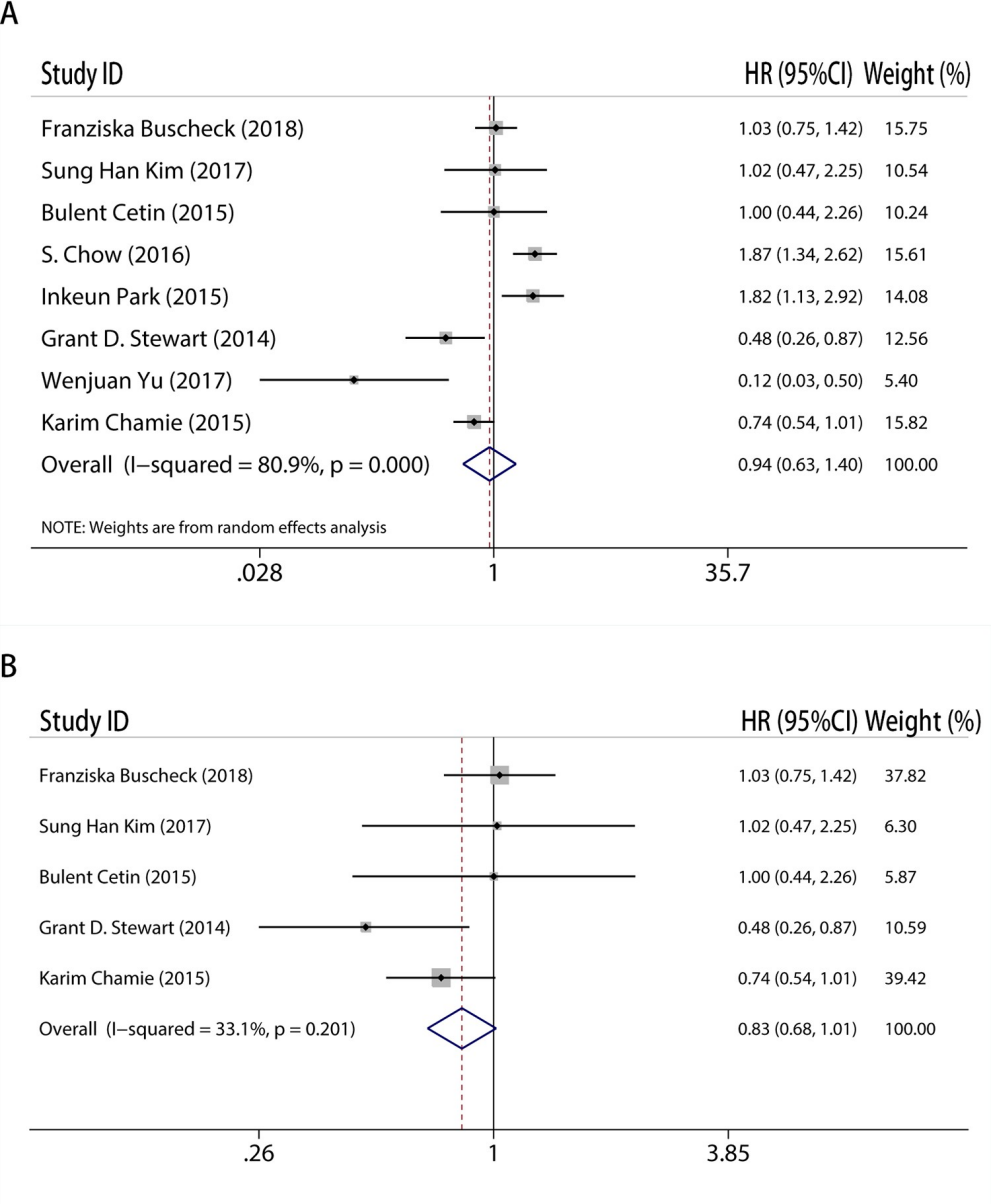
Meta-analysis of CAIX expression and overall survival on A, all new inclusion studies; B, by excluding the low quality score studies (quality score≤6).

In a similar manner, additional analyses were carried out for the relationship between CAIX expression and OS, with fifteen papers included. The researchers concluded that CAIX expression was not linked to OS (HR = 1.13, 95%CI: 0.82–1.56, I^2^ = 79.8%, P<0.05, Fig 3A). And notable heterogeneity made us have subgroup like Fig 2B. After removing low-quality research, the findings maintained the same (HR = 0.90, 95%CI: 0.75–1.07, I^2^ = 23.1%, P = 0.246, Fig 3B). As expected, the data supported that CAIX expression had no correlation with OS. The new study subgroup came to the same result (HR = 0.94, 95%CI: 0.63–1.40, I^2^ = 80.9%, P<0.05, Fig 4A). However, because substantial heterogeneity emerged, we carried an additional analysis in which low-quality papers (quality score≤6) were excluded in order to minimize heterogeneity. This led to a result which was consistent with the previous conclusion (HR = 0.83, 95%CI: 0.68–1.01, I^2^ = 33.1%, P = 0.201, Fig 4B).

### CAIX expression level was not associated to PFS in RCC

**Fig 5:**
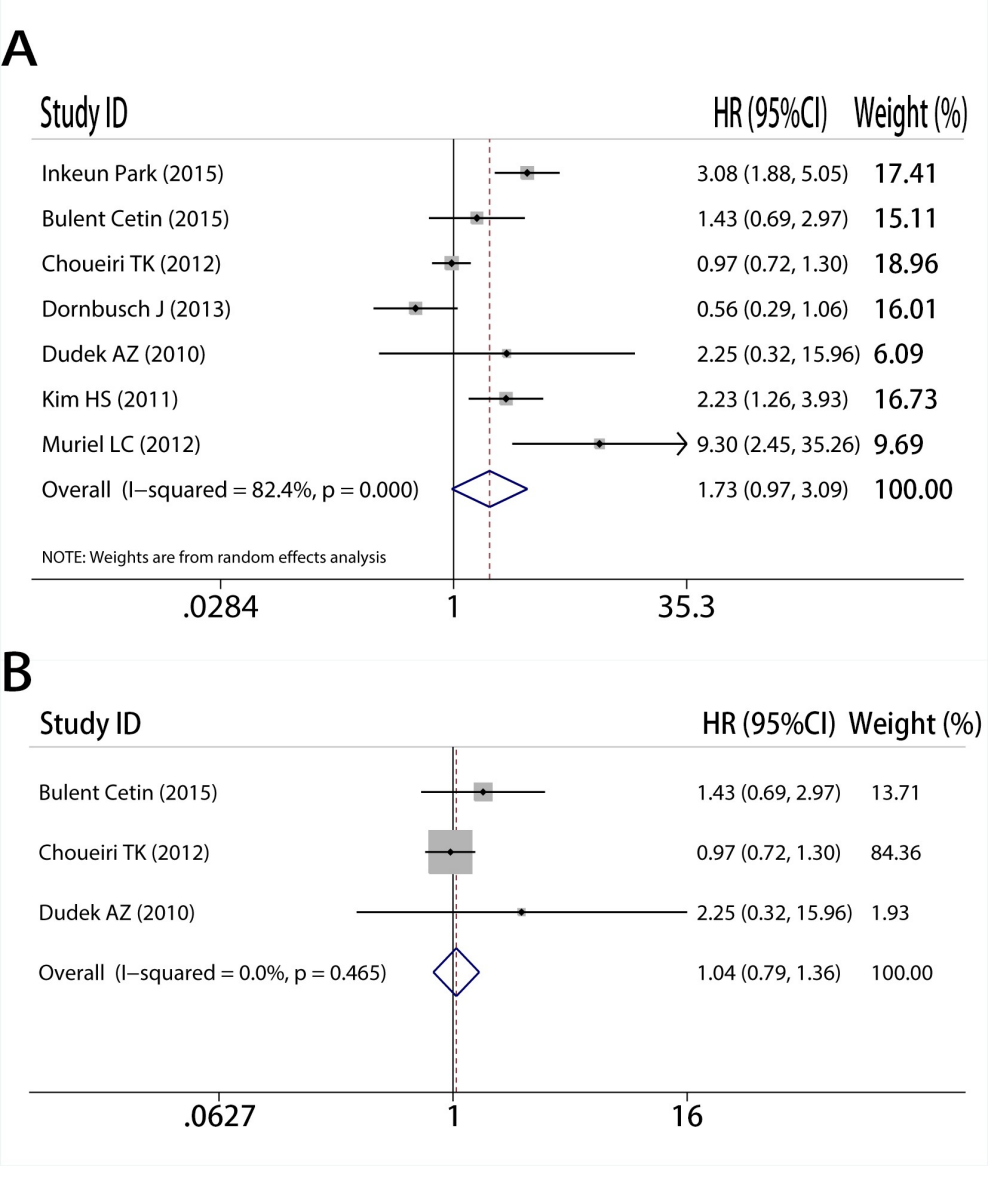
Meta-analysis of CAIX expression and progression-free survival on A, all inclusion studies; B, by excluding the low quality score studies (quality score≤6).

Due to the dearth of new research (just two), the analysis between CAIX and PFS was simplified. We only launched an analysis that included all trials and excluded low-quality studies. Took a total of 7 studies into consideration in the analysis (HR = 1.73, 95%CI: 0.97–3.09, I^2^ = 82.4%, P<0.05, Fig 5A), then the result indicated that CAIX expression was not associated with PFS. Then we need a subgroup to reduce heterogeneity, after excluding low-quality research, the conclusion held constant (HR = 1.04, 95%CI: 0.79–1.36, I^2^ = 0.0%, P = 0.465, Fig 5B).

### No relationships found between CAIX expression level and RFS in RCC

**Fig 6:**
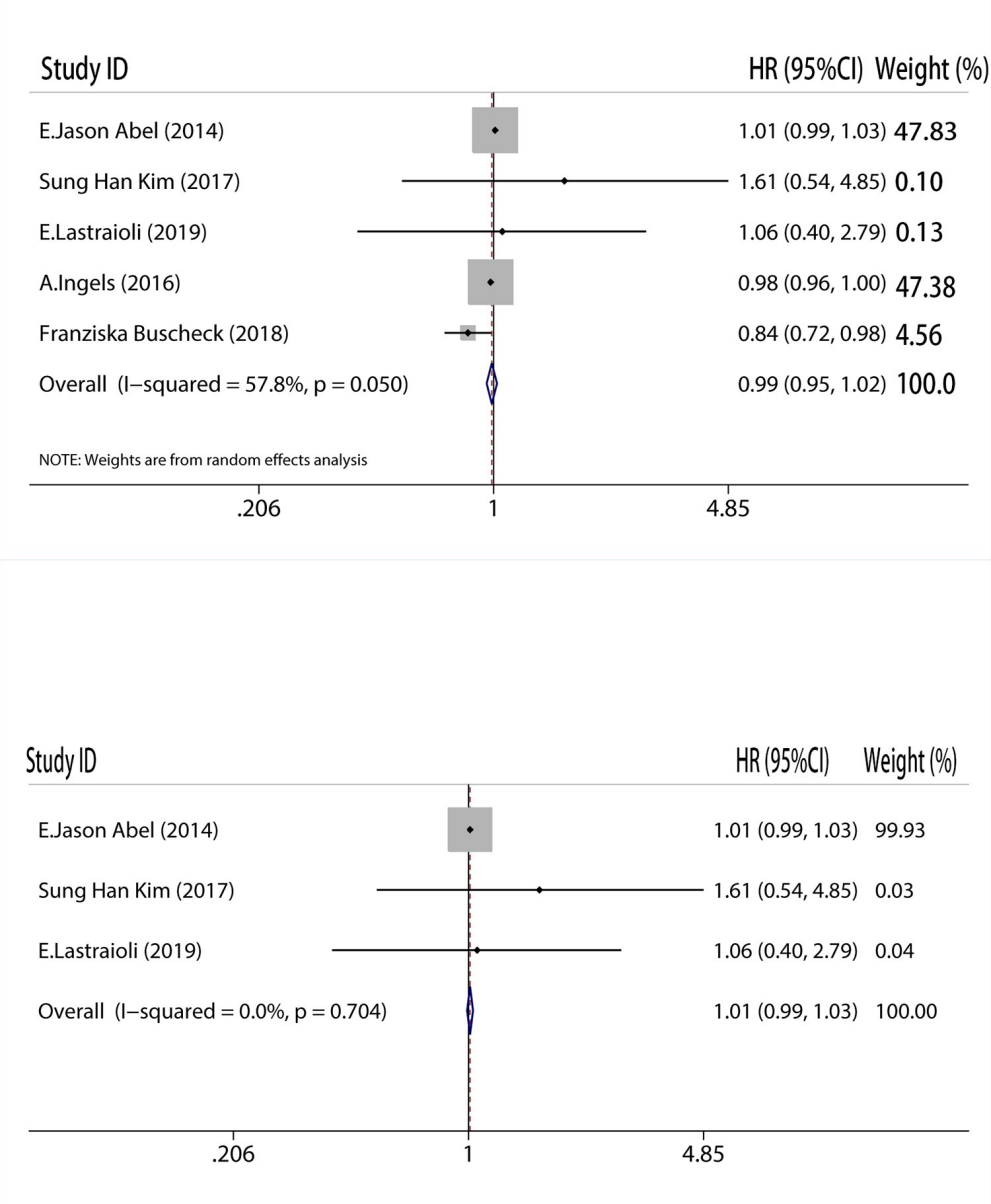
Meta-analysis of CAIX expression and recurrence-free survival on A, all inclusion studies; B, by excluding the low quality score studies (quality score≤7).

We also compared the relationship between CAIX and RFS. Also like other analysis, we detected no connection between CAIX and RFS. Because only new studies included had pertinent data, as a result, we discussed the impact that included all of the nascent research (HR = 0.99, 95%CI: 0.95–1.02, I^2^ = 57.8%, P = 0.050, Fig 6A). The heterogeneity still remained noteworthy; all of the studies captured more than 6 points, with five acquiring 8 points, and one receiving 7 points. As a result, a subgroup survey was applied, deleting the research with the lowest quality (HR = 1.01, 95%CI: 0.99–1.03, I^2^ = 0.00%, P = 0.704, Fig 6B). Whether Fig 6A or 6B, the hypothesis that CAIX expression has no relationship with RFS stayed consistent.

### Publication bias

**Fig 7:**
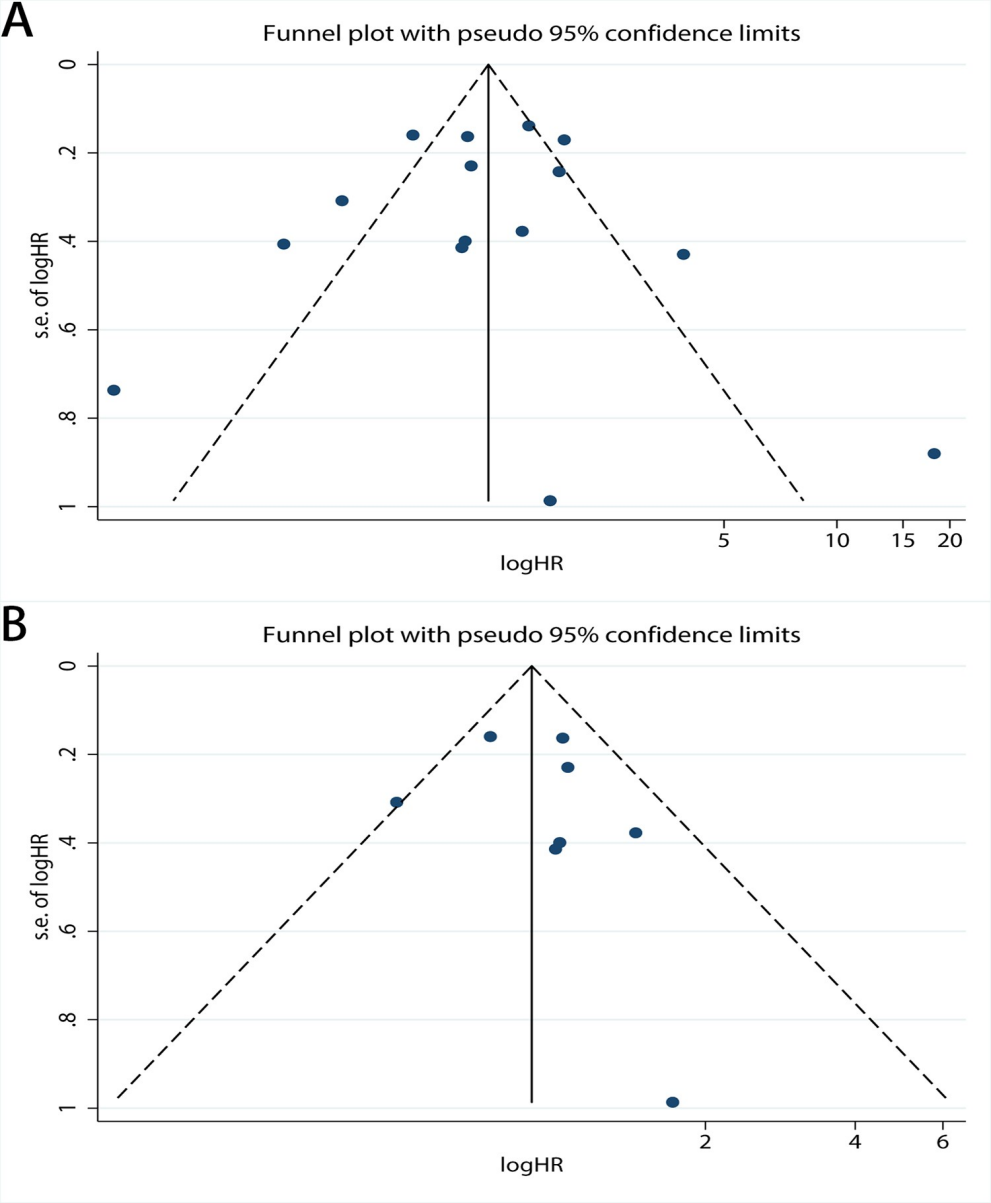
Funnel plot of CAIX expression and overall survival on A, all inclusion studies; B, by excluding the low quality score studies (quality score≤6).

**Fig 8:**
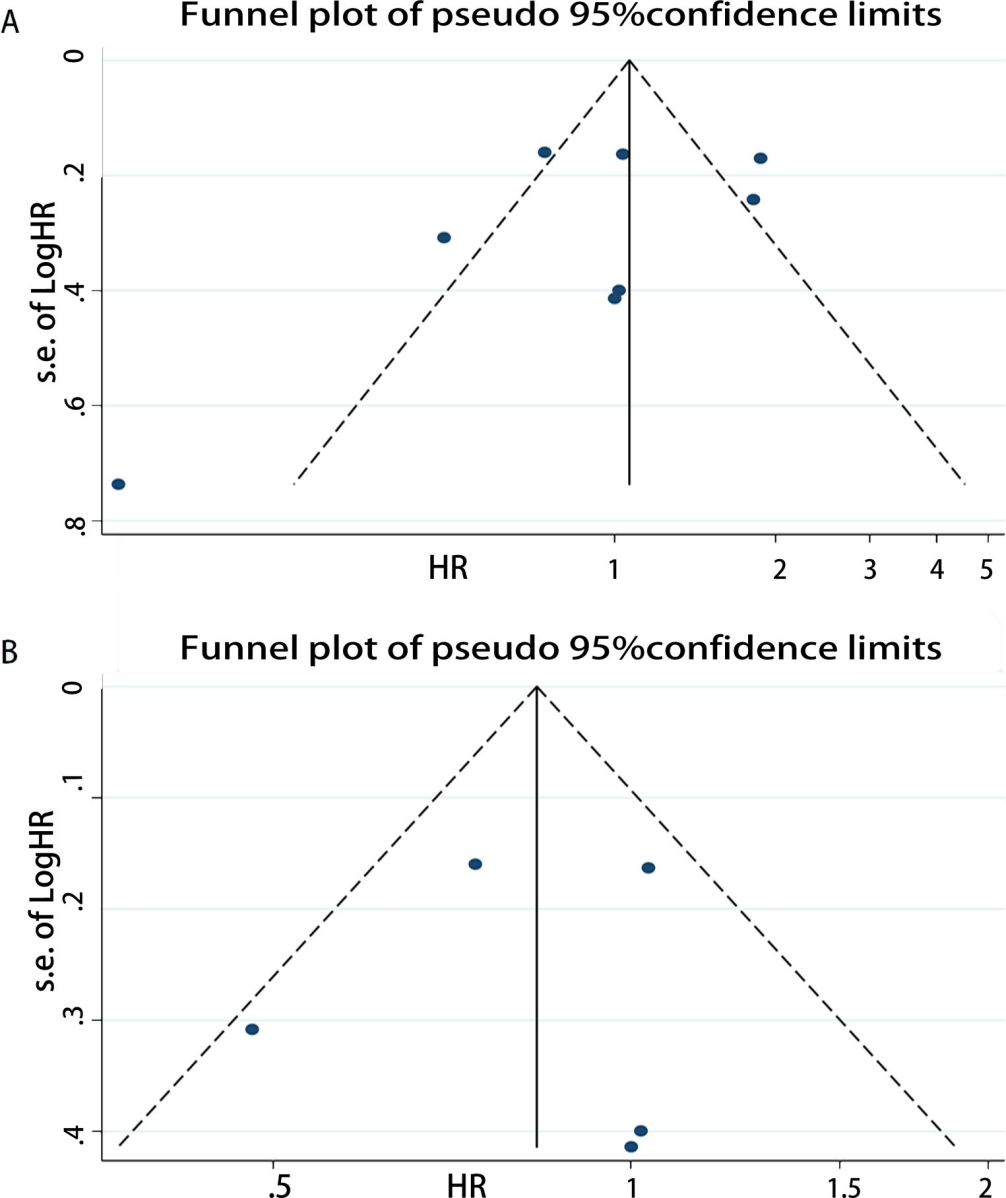
Funnel plot of CAIX expression and overall survival on A, all new inclusion studies; B, by excluding the low quality score studies (quality score≤6).

**Fig 9:**
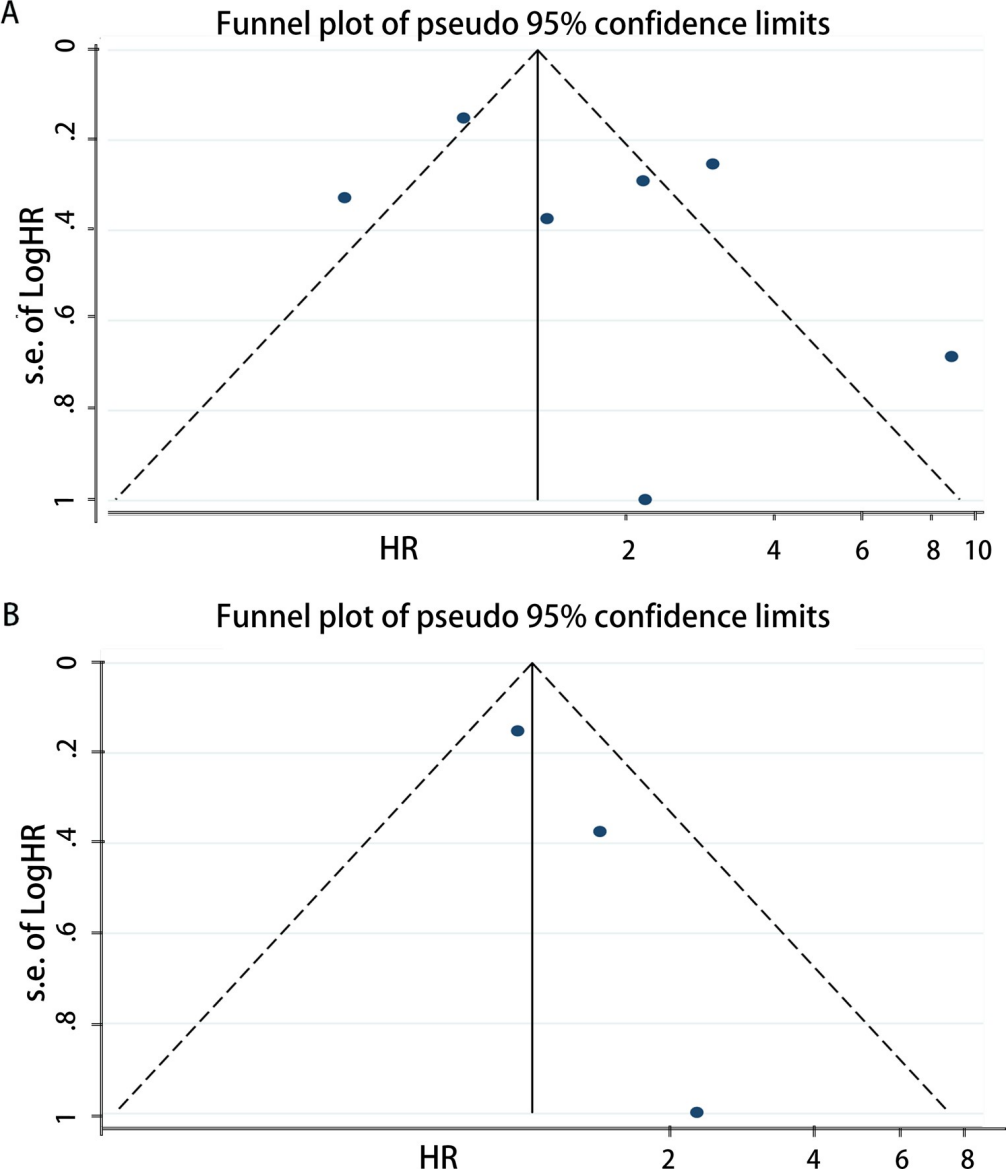
Funnel plot of CAIX expression and progression-free survival on A, all inclusion studies; B, by excluding the low quality score studies (quality score≤6).

**Fig 10:**
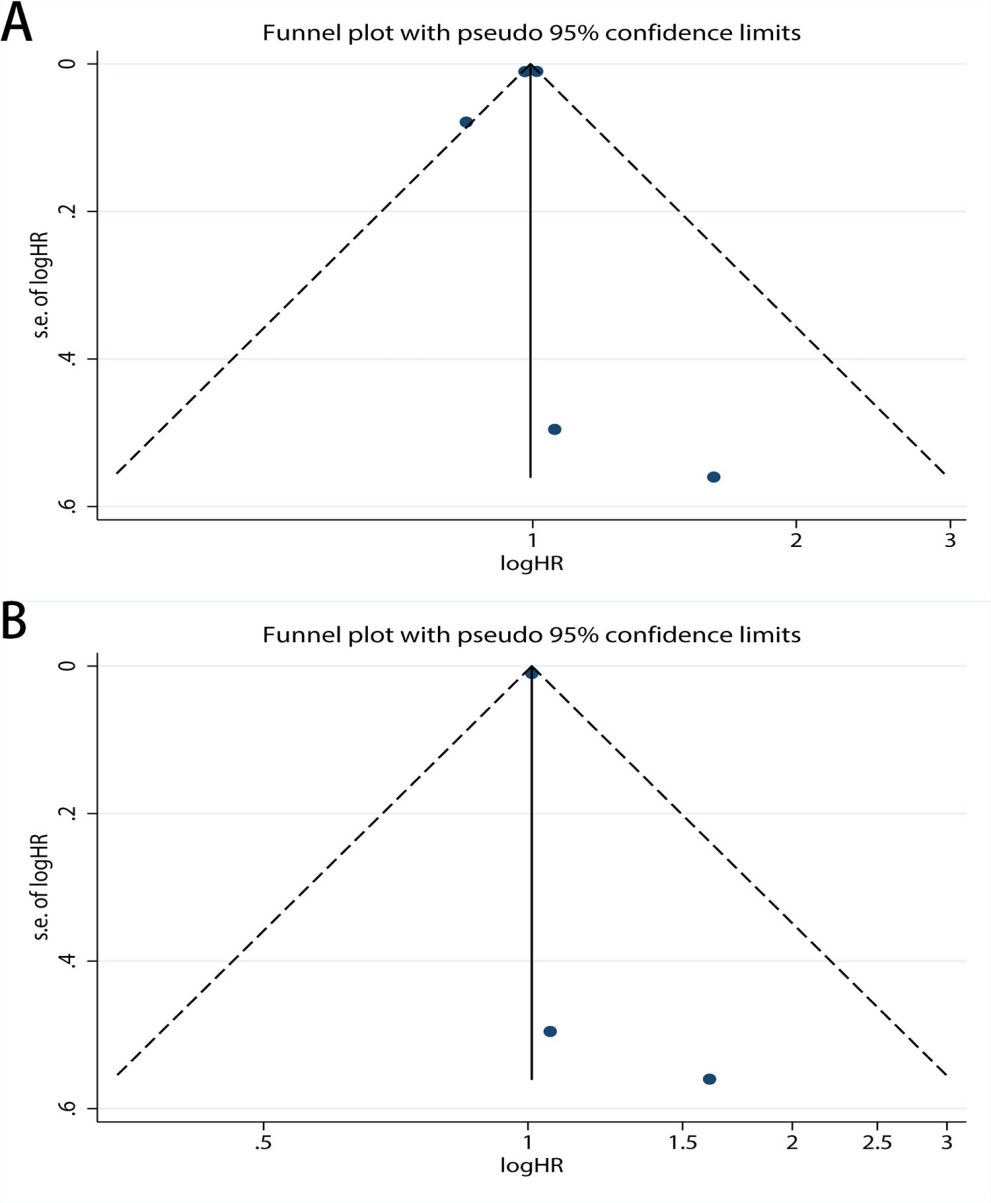
Funnel plot of CAIX expression and recurrence-free survival on A, all inclusion studies; B, by excluding the low quality score studies (quality score≤7).

**Fig 11:**
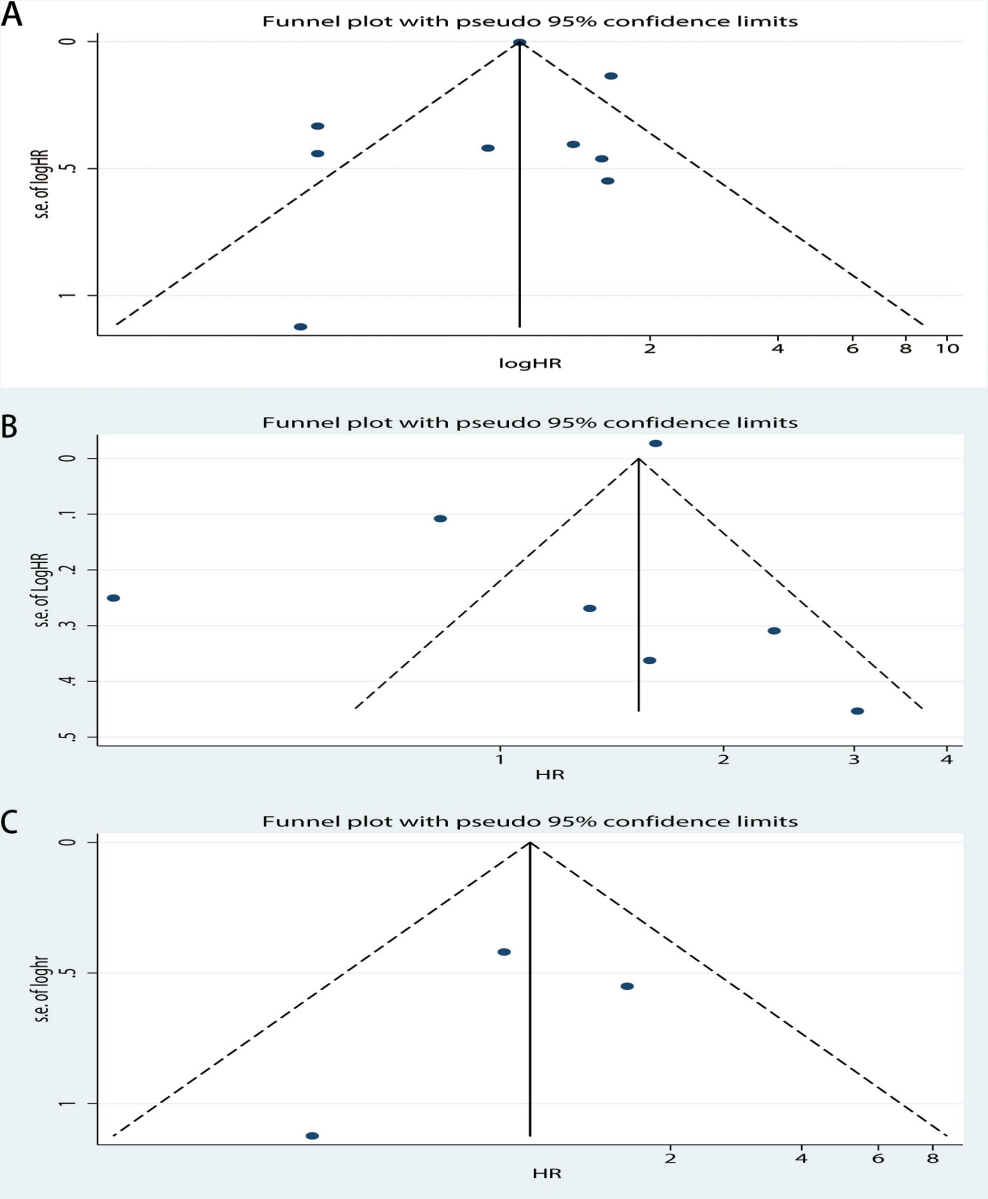
Funnel plot of CAIX expression and disease-free survival on A, all inclusion studies; B, by excluding the low quality score studies (quality score≤6); C, only including new studies (quality score≤6).

Publication bias was assessed for DSS, OS, PFS, and RFS respectively. For OS, the funnel plot of HR indicated some publication bias (Fig 7A). Publication bias was detected with a statistical test (Egger’s test = 0.04, Begg’s test = 0.921). After excluding low quality studies, publication bias reduced markedly (Fig 7B, Egger’s test = 0.160, Begg’s test = 0.902). The funnel plot of OS which only including new studies also revealed publication bias (Fig 8A, Egger’s test = 0.328, Begg’s test = 0.536) which smaller than Fig 7. And then we calculated publication bias of the subgroup which excluding low quality studies in all new research (Fig 8B, Egger’s test = 0.844, Begg’s test = 1.000). As for PFS, the funnel plot of HR also demonstrated a little publication bias (Fig 9A). Publication bias was consequently computed (Egger’s test = 0.308, Begg’s test = 0.764). Since the low-quality studies were eliminated, publication bias got accepted, just as it had before (Fig 9B, Egger’s test = 0.183, Begg’s test = 1.000). The funnel plot of RFS showed publication bias (Fig 10A), and the Egger’s test = 0.225, Begg’s test = 0.462. In the same way, there was a reduction in publication bias following the elimination of low quality studies (Fig 10B, Egger’s test = 0.710, Begg’s test = 1.000). Funnel plot of DSS indicated some bias (Fig 11A, Egger’s test = 0.771, Begg’s test = 0.466), then both the subgroup excluding low quality studies (Fig 11B, Egger’s test = 0.262, Begg’s test = 0.548) and the other which only including new studies (Fig 11C, Egger’s test = 0.666, Begg’s test = 1.000) had little publication bias. In summary, the publication bias in different endpoints can be accepted.

### TCGA data

**Fig 12:**
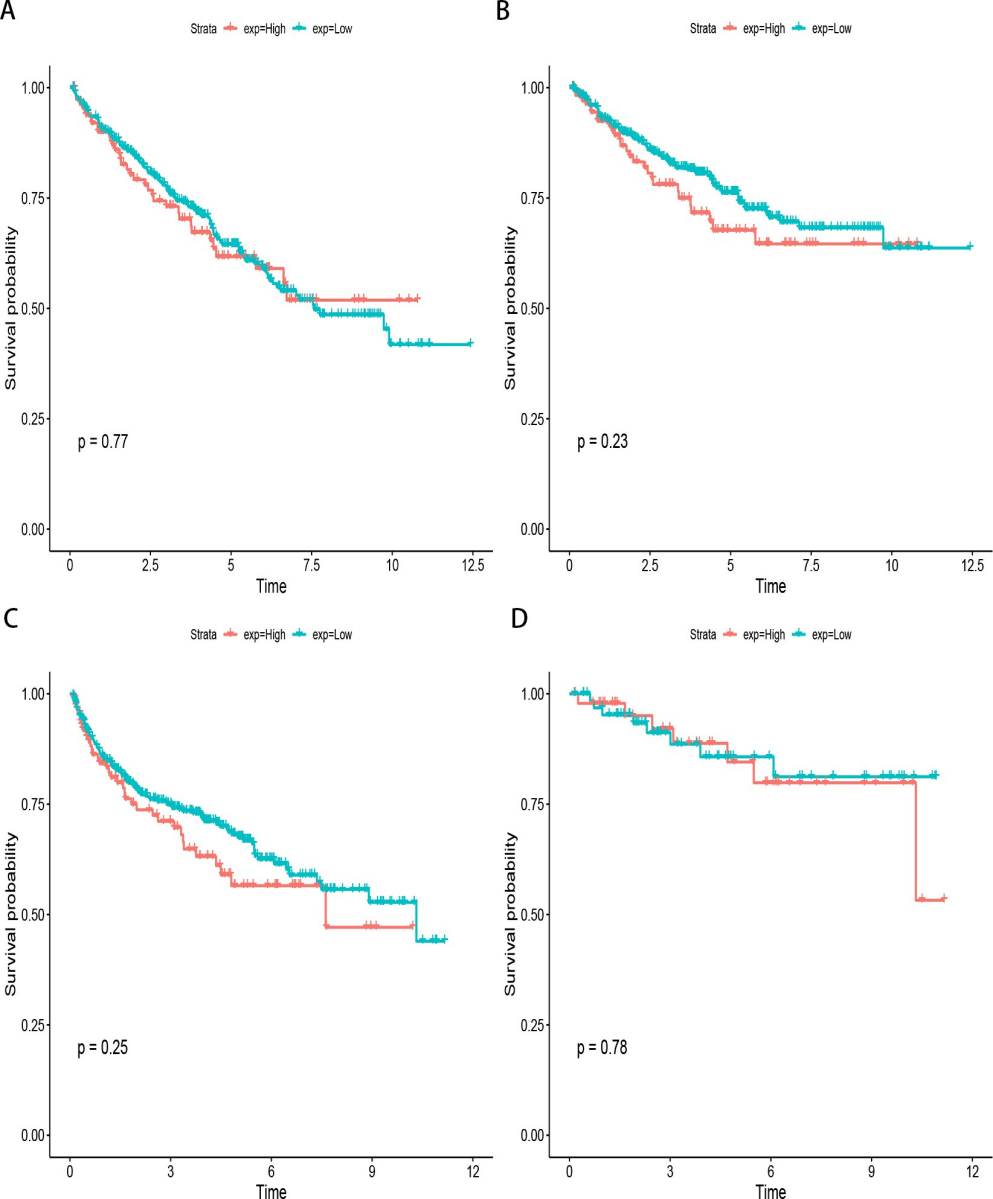
Bioinformatic analysis based on TCGA database. A, Kaplan-Meier survival analysis between CAIX expression and overall survival; B, Kaplan-Meier survival analysis between CAIX expression and disease-specific survival; C, Kaplan-Meier survival analysis between CAIX expression and progression-free interval; D, Kaplan-Meier survival analysis between CAIX expression and disease-free interval.

As a way to confirm the results above, we conducted a bioinformatic analysis of CAIX expression for survivals. These results matched those of a meta-analysis. Fig 12A–12D showed that all the endpoints had the same result, neither low nor high CAIX expression affected survival. Based on Kaplan-Meier analysis, CAIX expression did not associate with Overall survival (P = 0.77) (Fig 12A), disease-specific survival (P = 0.23) (Fig 12B), progression-free interval (P = 0.25) (Fig 12C) and disease-free interval (P = 0.78) (Fig 12D).

## Discussion

Clinical studies have explored the relationship between CAIX expression and treatment outcomes, which had multiple applications in tumor diagnosis, treatment and the prediction of clinical outcome. Some diseases, such as breast cancer can be effectively doped out by checking HER2 expression after radical surgery [53]. A lot of studies were searching for similar biomarkers to predict patients’ survival with RCC.

Our meta-analysis suggested that both low CAIX expression and high were not associated with survivals irrespectively of the endpoint evaluated. Association of CAIX expression on other tumors with patient prognosis has been validated. Among the many tumors in which CAIX was expressed are head and neck cancers [16], breast cancers [54–60], esophageal cancers [61, 62], pancreatic cancers [63–65], and soft tissue tumors [66, 67], whose worse prognosis was associated with the presence of CAIX. These results demonstrated the necessity of exploring the relationship between CAIX and RCC, and both their relatedness and lack of relationship. In addition to VHL genes and their associated proteins control CAIX expression, it has also been reported that PI3K and unfolded protein responses also regulated [68–70]. And this increased the CAIX expression and the uncertainty of prognosis with RCC. RCC was not the only cancer where CAIX level did not correlate with patient prognosis, as research on ovary cancer [30, 31, 33], bladder cancer [71], and cervical cancer [72–74] followed the similar result. CAIX correlated with the survival of some tumors, but not all. Like brain cancer, the results of studies varied; 9 papers concluded that CAIX was linked with OS [75–83], while 3 papers [84–86] around PFS found it not to be. Contradictory results may be due to different cut-off values and different manufacturers’ reagents were used in immunohistochemical staining.

To further explain our results, we examined the upstream genes and related regulators of CAIX expression in RCC. RCC was distinguished from other cancers by a somatic mutation of the VHL gene which occurred frequently [87, 88], in most RCC especially clear cell subtype (ccRCC). Our results may have been affected by this factor in contrast with other tumors expressed CAIX. When tumor is in hypoxic conditions, HIF-1α (HIF has two parts, a constitutive β-subunit and an oxygen-sensitive α subunit [15]) can not be hydroxylated by prolyl hydroxylase domain proteins and bounded by pVHL [89] because of Von Hippel-Lindau (VHL) gene mutation, then HIF-1α can not be in subsequent degradation by 26S proteasome [90–93]. This position leads to the durative accumulation and activation of HIF-1α [94]. In abnormal oxygen statuses and acidic conditions, CAIX expression is mediated by the HIF transcriptional complex [95]. In the literature, the HIF-1α non-oxygen concentration dependence accumulation because of VHL gene mutations leads to an abnormal increase in CAIX so that it can not reflect real hypoxic status, and then it is accordant that the results of our analysis show no correlation between CAIX level and prognosis.

Numerous studies [96–103] manifested the dependency of CAIX expression on HIF-1α under hypoxic conditions. And showed the HIF-1-responsive element HRE was localized next to CAIX transcription start site [30], suggesting that CAIX was a HIF-1 downstream target gene. In summary, this behavior of CAIX expression in RCC can be attributed to the pVHL which prevented proteasomal degradation of CAIX upon normoxia and expressed without hypoxia [87, 104]. To sum up, VHL gene mutation led to pVHL deletion, which can not degrade HIF-1α, resulted in abnormal accumulation of HIF-1α. Under normal or hypoxic conditions, HIF-1α can regulate CAIX overexpression, which can not reflect tumor hypoxia by testing CAIX expression level, and can not predict the prognosis of patients. This mechanism contributed to the independence between CAIX expression and real hypoxic status and then indirectly estranged from survival rates, so it explained our results that CAIX expression was not associated with several endpoints. By going through aforementioned studies, we can explain the result of meta-analysis and bioinformatic analysis based on TCGA database, and understood why CAIX expression was not associated with different endpoints.

A meta-analysis [12] carried out in 2014 connected low expression of CAIX to poor DSS, OS and PFS. It seemed contrary to the functional mechanism of CAIX. Although Several studies have also suggested a positive correlation between CAIX levels and the IL-2 response of patients with RCC undergoing treatment [18–20]. In this case, it can only be stated that CAIX may be used as an effective therapeutic target, but it cannot be determined whether its expression level has a connection with patient survival(especially when no treatment aiming at this target). Some studies found different or even opposite effects on different subtypes [19, 44, 95], for instance, a study reported that in ccRCC, high CAIX expression had more favorable prognosis in OS and RFS, while in papillary RCC (pRCC) the result was opposite, although the P value did not reach the level of statistical significance in OS (P = 0.1645 for ccRCC, P = 0.3861 for pRCC). We do not evaluate the association between CAIX expression and T stage, N stage, M stage and Furhman grade since few new studies after 2014 reported complete data about them, and the association between CAIX level and T stage has been demonstrated irrelevant [12]. According to these results, CAIX expression was neither a suppressing nor a promoting factor for patients with RCC. No matter what kind of analysis we used, all of them led to the conclusion that CAIX was an unsuitable biomarker for predicting the prognosis of RCC.

Compared with the meta analysis [12] a few years ago, we have included more literature and drawn more scientific conclusions. We assessed the relevance of CAIX to RFS in addition to DSS,OS and PFS. In order to make the conclusions more reliable, we used bioinformatic analysis to verify the conclusions. And similar results were also obtained in bioinformatic analysis.

We also have some limitations, for instance, the level of evidence provided by observational studies was less than that provided by randomized controlled trials; and most of the studies included in our meta-analysis were retrospective studies. In our research, there was significant heterogeneity among 27 included studies. And because of the number of including studies, the funnel plot of publication bias may have very low power to distinguish chance from real asymmetry. Compared with the former analysis [12], we did not limit the literature language so we get a more complete literature search [52]. Heterogeneity may have been caused by some factors such as patients coming from different countries with different tumor stages and histological types, cut-off values, the therapy methods used, follow-up time, different sources and dilutions of primary antibodies. For the reduction of heterogeneity, only IHC-based studies evaluating CAIX expression levels were included. Further, it was necessary to take the publication bias into account. For OS, the result exhibited publication bias (Figs 7A and 8A). However, when the low quality studies were excluded, there existed little publication bias (Figs 7B and 8B). Furthermore, there was no significant publication bias for RFS and DSS (Figs 10A and 11A), that indicated the analyses were feasible and the results were credible. Another limitation was the process of data extraction. The data was calculated by using survival curves when the study did not provide HR and SE directly, this process also introduced a potential source of bias.

In conclusion, Our meta-analysis indicated that no difference found between low and high CAIX expression detected by IHC was associated with poor DSS, OS,PFS and RFS in patients with RCC.

## Supporting information

S1 Checklist PRISMA checklist for this meta-analysis. (DOC)

S1 Table Newcastle–Ottawa quality assessment scale. (PDF)

S2 Table Quality assessment of each study included. (TIF)

S1 Data Raw data and the final data for survival outcome. (DOC)
