# Correction: A systematic review verified by bioinformatic analysis based on TCGA reveals week prognosis power of CAIX in renal cancer

**DOI:** 10.1371/journal.pone.0315303

**Published:** 2024-12-05

**Authors:** Zikuan Zhang, Bo Wu, Yuan Shao, Yongquan Chen, Dongwen Wang

There are errors in the author affiliations. The correct affiliations are as follows:

Zikuan Zhang^1^, Bo Wu^1,2^, Yuan Shao^1^, Yongquan Chen^1^, Dongwen Wang^1,3^

**1.** Shanxi Medical University, Taiyuan, Shanxi, China **2.** Department of urology, First hospital of Shanxi Medical University, Taiyuan, Shanxi, China **3.** National Cancer Center/National Clinical Research Center for Cancer/Cancer Hospital & Shenzhen Hospital, Chinese Academy of Medical Sciences and Peking Union Medical College, Shenzhen, Guangdong, China
