# Supplementary figures and images for: A systematic review verified by bioinformatic analysis based on TCGA reveals week prognosis power of CAIX in renal cancer
Source: PLoS One. 2022 Dec 21;17(12):e0278556. doi: 10.1371/journal.pone.0278556 (PMC9770376; doi:10.1371/journal.pone.0278556)

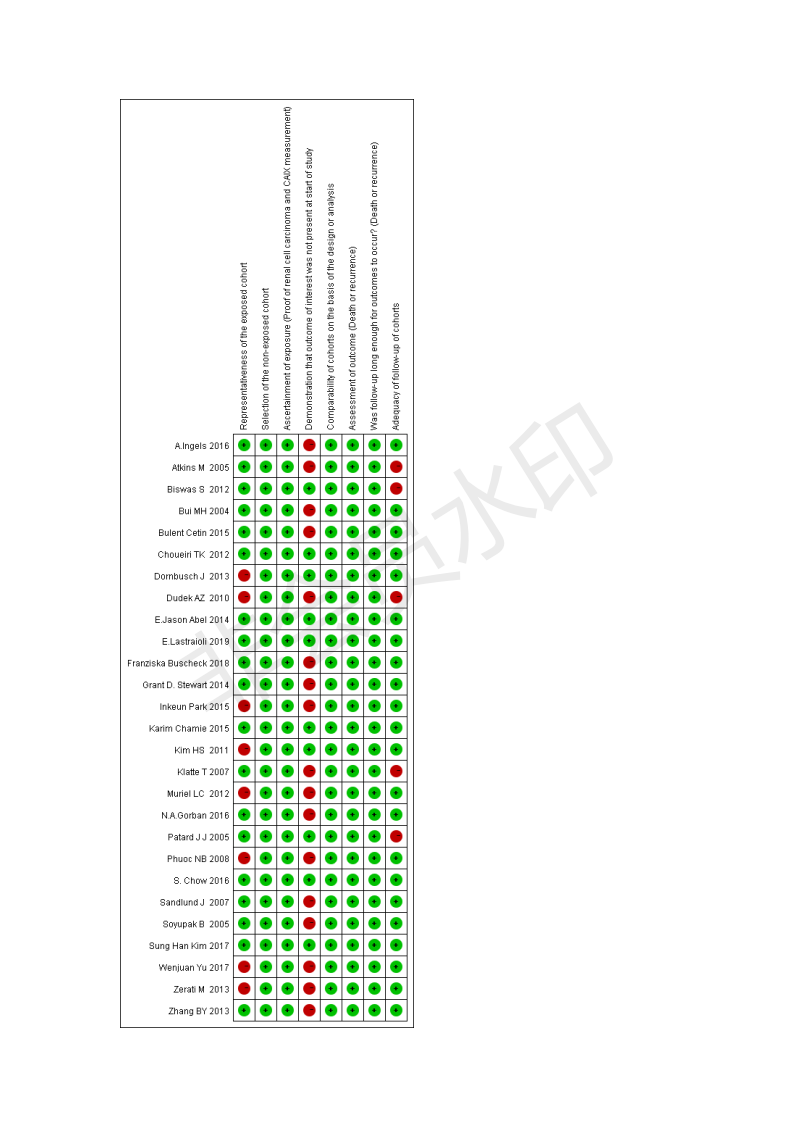

Supplement: S2 Table — (TIF) [file pone.0278556.s003.tif]
