# Supplementary material for: A systematic review verified by bioinformatic analysis based on TCGA reveals week prognosis power of CAIX in renal cancer
Source: PLoS One. 2022 Dec 21;17(12):e0278556. doi: 10.1371/journal.pone.0278556 (PMC9770376; doi:10.1371/journal.pone.0278556)
Supplement: S1 Data — (DOC) [file pone.0278556.s004.doc]

| First author | year | Survival analysis | Survival data extraction | HR | 95%CI |
| --- | --- | --- | --- | --- | --- |
| Bui MH | 2004 | univariate | curves | 1.54 | 0.62-3.79 |
| Patard JJ | 2005 | multivariate | reported | 0.33 | 0.14-0.79 |
| Klatte T | 2007 | multivariate | reported | 0.987 | 0.981-0.993 |
| Sandlund J | 2007 | multivariate | calculated | 1.32 | 0.60-2.93 |
| Phuoc NB | 2008 | multivariate | reported | 0.33 | 0.172-0.634 |
| Zhang BY | 2013 | Univariate | reported | 1.62 | 1.24-2.11 |
| Sung Han Kim | 2017 | univariate | reported | 1.59 | 0.54-4.65 |
| 1. A. Gorban | 2016 | univariate | calculated | 0.831 | 0.365-1.892 |
| E.Lastraioli | 2019 | univariate | calculated | 0.301 | 0.033-2.703 |

1.Data extraction for disease-specific survival

2.Data extraction for overall survival

3.Data extraction for progression-free survival

| First author | year | Survival analysis | Survival data extraction | HR | 95%CI |
| --- | --- | --- | --- | --- | --- |
| Atkins M | 2005 | Univariate | calculated | 1.51 | 1.15-1.98 |
| Soyupak B | 2005 | Univariate | reported | 3.90 | 1.68-9.05 |
| Dudek AZ | 2010 | Univariate | Calculated | 1.72 | 0.25-11.95 |
| Biswas S | 2012 | Multivariate | reported | 1.45 | 0.69-3.03 |
| Muriel LC | 2012 | Univariate | Calculated | 18.17 | 3.24-101.98 |
| Dornbusch J | 2013 | Univariate | reported | 0.335 | 0.151-0.742 |
| Zerati M | 2013 | Univariate | calculated | 1.06 | 0.68-1.67 |
| Grant D. Stewart | 2014 | univariate | reported | 0.48 | 0.26-0.87 |
| Bulent Cetin | 2015 | univariate | reported | 1.001 | 0.445-2.255 |
| Inkeun Park | 2015 | multivariate | reported | 1.818 | 1.131-2.922 |
| Karim Chamie | 2015 | multivariate | reported | 0.74 | 0.54-1.01 |
| S. Chow | 2016 | univariate | calculated | 1.874 | 1.342-2.619 |
| Sung Han Kim | 2017 | univariate | reported | 1.02 | 0.47-2.25 |
| Wenjuan Yu | 2017 | univariate | calculated | 0.118 | 0.028-0.503 |
| Franziska Buscheck | 2018 | univariate | calculated | 1.035 | 0.752-1.425 |

| First author | year | Survival  analysis | survival data extraction | HR | 95%CI |
| --- | --- | --- | --- | --- | --- |
| Dudek AZ | 2010 | Univariate | calculated | 2．25 | 0．32-15．96 |
| Kim HS | 2011 | Univariate | calculated | 2.23 | 1.26-3.93 |
| Muriel LC | 2012 | Univariate | calculated | 9.30 | 2.45-35.26 |
| Choueiri TK | 2012 | Univariate | calculated | 0.97 | 0.72-1.30 |
| Dornbusch J | 2013 | Univariate | reported | 0.559 | 0.294-1.062 |
| Bulent Cetin | 2015 | univariate | reported | 1.428 | 0.686-2.970 |
| Inkeun Park | 2015 | multivariate | reported | 3.079 | 1.877-5.051 |

4.Data extraction for recurrence-free survival

| First author | year | Survival  analysis | survival data extraction | HR | 95%CI |
| --- | --- | --- | --- | --- | --- |
| E.Jason Abel | 2014 | univariate | reported | 1.01 | 0.99-1.03 |
| A.Ingels | 2016 | univariate | reported | 0.98 | 0.96-1.00 |
| Sung Han Kim | 2017 | univariate | reported | 1.61 | 0.54-4.85 |
| Franziska | 2018 | univariate | calculated | 0.84 | 0.72-0.98 |
| E. Lastraioli | 2019 | univariate | calculated | 1.06 | 0.40-2.79 |
